# Telemedicine-Guided Two-Incision Lower Leg Fasciotomy Performed by Combat Medics During Tactical Combat Casualty Care: A Feasibility Study

**DOI:** 10.1093/milmed/usad364

**Published:** 2023-09-12

**Authors:** P W Stark, B L S Borger van der Burg, O J F van Waes, T T C F van Dongen, 1 Wouter, Marnalg Casper, R Hoencamp

**Affiliations:** Trauma Research Unit, Department of Surgery, Erasmus University Medical Center, Rotterdam, Zuid-Holland 3015 GD, The Netherlands; Department of Surgery, Alrijne Hospital, Leiderdorp, Zuid-Holland 2353 GA, The Netherlands; Department of Surgery, Alrijne Hospital, Leiderdorp, Zuid-Holland 2353 GA, The Netherlands; Trauma Research Unit, Department of Surgery, Erasmus University Medical Center, Rotterdam, Zuid-Holland 3015 GD, The Netherlands; Defense Healthcare Organization, Ministry of Defense, Den Haag, Zuid-Holland 2511 CB, The Netherlands; Department of Surgery, Alrijne Hospital, Leiderdorp, Zuid-Holland 2353 GA, The Netherlands; Defense Healthcare Organization, Ministry of Defense, Den Haag, Zuid-Holland 2511 CB, The Netherlands; Defense Healthcare Organization, Ministry of Defense, Den Haag, Zuid-Holland 2511 CB, The Netherlands; Defense Healthcare Organization, Ministry of Defense, Den Haag, Zuid-Holland 2511 CB, The Netherlands; Trauma Research Unit, Department of Surgery, Erasmus University Medical Center, Rotterdam, Zuid-Holland 3015 GD, The Netherlands; Department of Surgery, Alrijne Hospital, Leiderdorp, Zuid-Holland 2353 GA, The Netherlands; Defense Healthcare Organization, Ministry of Defense, Den Haag, Zuid-Holland 2511 CB, The Netherlands

## Abstract

**Introduction:**

During tactical combat casualty care, life- and limb-saving procedures might also be performed by combat medics. This study assesses whether it is feasible to use a head-mounted display (HMD) to provide telemedicine (TM) support from a consulted senior surgeon for combat medics when performing a two-incision lower leg fasciotomy.

**Materials and Methods:**

Nine combat medics were randomized into groups to perform a two-incision lower leg fasciotomy. One group used the Vuzix M400 and the second group used the RealWear HMT-1Z1. A third, control, group received no guidance. In the Vuzix M400 group and RealWear HMT-1Z1 group, a senior surgeon examined the results after the two-incision lower leg fasciotomy was finished to assess the release of compartments, possible collateral damage, and performance of the combat medics. In the control group, these results were examined by a surgical resident with expertise in two-incision lower leg fasciotomies. The resident’s operative performance questionnaire was used to score the performance of the combat medics. The telehealth usability questionnaire was used to evaluate the usability of the HMDs as perceived by the combat medics.

**Results:**

Combat medics using an HMD were considered competent in performing a two-incision lower leg fasciotomy (Vuzix: median 3 [range 0], RealWear: median 3 [range 1]). These combat medics had a significantly better score in their ability to adapt to anatomical variances compared to the control group (Vuzix: median 3 [range 0], RealWear: median 3 [range 0], control: median 1 [range 0]; *P* = .018). Combat medics using an HMD were faster than combat medics in the control group (Vuzix: mean 14:14 [SD 3:41], RealWear: mean 15:42 [SD 1:58], control: mean 17:45 [SD 2:02]; *P* = .340). The overall satisfaction with both HMDs was 5 out of 7 (Vuzix: median 5 [range 0], RealWear: median 5 [range 1]; *P* = .317).

**Conclusions:**

This study shows that it is feasible to use an HMD to provide TM support performance from a consulted senior surgeon for combat medics when performing a two-incision lower leg fasciotomy. The results of this study suggest that TM support might be useful for combat medics during tactical combat casualty care when performing life- and limb-saving procedures.

## INTRODUCTION

Telemedicine (TM), literally translated as “healing at a distance” (from Latin “medicus” and Greek “tele”), has been used for over a hundred years.^1^ The information-sharing capacity of TM enables access to consulted senior surgeons to support medical decision-making on scene or during tactical combat casualty care.^2^ Many different techniques are available to support TM, including head-mounted displays (HMDs).^3^ HMDs are wearables that present data onto lenses and record images or videos through a front-facing camera^3^ by which a consulted senior surgeon can observe life- and limb-saving procedures and use telestration for precise instructions, as an alternative to the need for physical presence to supervise. Telestration is defined as a technique for enabling annotations over an image or video.^4^ Google Glasses (Google, Mountain View, California, United States) have been used in surgery before.^5^ However, these HMDs are no longer commercially available. More recently developed HMDs from Vuzix (Rochester, New York, United States) and RealWear (RealWear, Vancouver, Washington, United States) are also suitable for application in surgery, based on functionalities and software options.

The tactical combat casualty care guidelines were developed to provide guidance for medical care on the battlefield and in austere environments.^6^ Type of injury, location of the injured combatant, and the tactical situation determine in which phase of tactical combat casualty care certain medical interventions can be performed. Life- and limb-saving surgery interventions are preferably performed by forward surgical teams or by experienced surgical experts in support hospitals.^7^ Because of the dynamics and hostile environment in armed conflicts, evacuation to a higher level of care is not always possible. In these cases, parts of life- and limb-saving interventions can also be performed by non-surgically trained military medical personnel (combat medics) during tactical combat casualty care. The performance of life- and limb-saving procedures during tactical combat casualty care by combat medics will lead to better outcomes and greater survivability of combatants.^8^ TM support from a consulted senior surgeon might be the only option to safely perform life- and limb-saving surgery procedures during tactical combat casualty care by combat medics.

Compartment syndrome of the extremities may occur acutely or as a chronic syndrome. Acute compartment syndrome is a limb-threatening condition, which can be caused by penetrating injuries, such as gunshot wounds, crush injuries, blast injuries, vascular injuries, and fractures.^9^ Despite its limitations, clinical assessment is the most important diagnostic modality to diagnose acute compartment syndrome.^10^ Various diagnostic techniques may help to support this clinical assessment, but there is no strong evidence to support the use of these techniques during tactical combat casualty care.^11^ Timely performance of a two-incision lower leg fasciotomy is an example of a life- and limb-saving intervention, which is performed when acute compartment syndrome is suspected in patients after sustaining fractures, traumatic blast injuries, penetrating injuries, or crush injuries. When fasciotomy is described in the remainder of this article, this refers to a two-incision lower leg fasciotomy. Incomplete or delayed fasciotomies are associated with nerve and/or vascular damage and muscle necrosis, which can lead to loss of function, major amputation, and mortality.^12^ Proficiency in performing a fasciotomy can be difficult to acquire and retain, even for practicing surgeons.^13^

Sparse evidence suggests that fasciotomies can be successfully performed in austere environments by combat medics with TM support.^14–17^ However, based on these reports,^14–17^ no conclusions can be drawn whether it is feasible to use an HMD to provide TM support for combat medics when performing a fasciotomy. Currently, there is no legislation that describes the competency of combat medics to perform life- and limb-saving procedures. TM could help bridge the gap between the lack of legislation and the need for the ability to perform complex life- and limb-saving procedures.

The primary objective of this study was to assess whether it is feasible to use an HMD to provide TM support from a consulted senior surgeon for combat medics when performing a fasciotomy. The secondary objective was to investigate the satisfaction, intent to continue use, and perceived appropriateness of using an HMD during the performance of a fasciotomy by combat medics. Technical factors involving HMD usage were also assessed.

## MATERIALS AND METHODS

This study is a feasibility study with an experimental laboratory design, which was conducted in the Skillslab & Simulation Center of Erasmus University Medical Center, Rotterdam, the Netherlands. The AnubiFiX-embalmed postmortem human specimens^18,19^ used for the interventions were donated for scientific research and medical training at the Anatomy Department of Skillslab, Rotterdam, and were part of a national body donation program approved by Dutch law and regulations. A protocol for this study was reviewed and approved by the Dutch Ministry of Defense and the Institutional Review Board of Alrijne Hospital, the Netherlands (NWMO 17-15, 17.409rt.tk). All participants completed an informed consent to participate in this study.

### Participants

Two senior surgeons and a surgical resident with expertise in performing fasciotomies were participated as supervisors in this study. Participants were selected from a group of non-surgically trained military medical personnel, in this study combat medics (combatants with add-on training for acute care medicine).

### Hardware and Software

**FIGURE 1.**
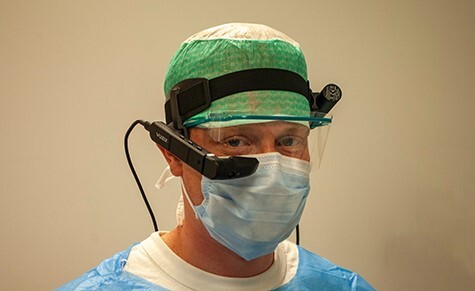
An example of a combat medic wearing the Vuzix M400.

**FIGURE 2.**
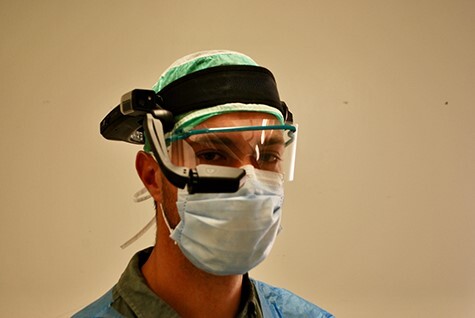
An example of a combat medic wearing the RealWear HMT-1Z1.

The Vuzix M400 (Fig. 1; Vuzix, Rochester, New York, United States) and the RealWear HMT-1Z1 (Fig. 2; RealWear, Vancouver, Washington, United States) were selected to conduct the study. The selection was based on hardware and software characteristics. Both devices are water resistant and dust tight. Connectivity options range from Bluetooth and Wi-Fi to Global Positioning Systems, 4G, and satellite connection. Two MacBook Air 2017 devices (Apple, Cupertino, California, United States) were used to observe and telestrate the fasciotomies performed by the combat medics. The HMDs and MacBooks were connected to a Wi-Fi Protected Access 2 encrypted Wi-Fi network. Enovation Zaurus software (Zaurus, Alkmaar, the Netherlands) was installed on both MacBooks and on the HMDs used. Enovation Zaurus software is certified with ISO 27001 and NEN 7510 standards for information security in health care, ISO 27701 privacy standard, and the ISO 9001 quality standard.

### Study Procedure

At the start of the study, all combat medics attended a standardized concise lecture as part of a formalized curriculum. This curriculum was based on the core principles of microteaching and consisted of instructions on the basic anatomy of the lower leg, indications for a fasciotomy, and surgical techniques for performing a fasciotomy. The lecture also included details and instructions for the use of the HMD. The study was conducted in three 30-min time blocks, with three combat medics per time block. After a short setup period of 5 min, the combat medics performed a fasciotomy on an AnubiFiX^®^-embalmed postmortem human leg^18,19^ in the operating room (OR), which is based in the Skillslab & Simulation Center.

At the start of the study, the combat medics were randomly assigned to groups performing a fasciotomy while using an HMD to receive remote support from a consulted senior surgeon, who was located in another room without the possibility of direct contact with the combat medics. Combat medics could also be randomly assigned to the control group, in which they performed a fasciotomy without guidance. In the control group, a surgical resident solely observed the intervention in the OR, with respect to safety, instrument use, and tissue handling, and scored the performance of the combat medics.

### Study Parameters

The main study parameter of this study was the performance of combat medics. The consulted senior surgeon examined the AnubiFiX-embalmed postmortem human leg^18,19^ after the fasciotomy was finished to assess the release of compartments and possible collateral damage. In the control group, these results were examined by the surgical resident. The performance of the combat medics was also evaluated by the resident’s operative performance questionnaire (ROPQ).^20^ The ROPQ was completed after each fasciotomy by the consulted senior surgeon. The ROPQ is a validated assessment tool and consists of nine questions, with scores ranging from 1 to 5, where score 1 represents the poorest and 5 the best performance. If the consulted senior surgeon was unable to assess a certain question, it was scored with zero. Some questions had additional information to support scoring.

The secondary study parameter of this study was the usability of the HMD used, which was assessed with the telehealth usability questionnaire (TUQ).^21^ This is a validated tool specifically developed to evaluate the usability of telehealth implementation and services. The TUQ has 21 items that are based on six components of usability: Usefulness (three items), ease of use and learnability (three items), interface quality (four items), interaction quality (four items), reliability (three items), and satisfaction and future use (four items).^22^ Each item utilizes a 7-point Likert scale to measure a component of usability, with the value of 1 for least usable and 7 for most usable. The TUQ was completed after each fasciotomy by all combat medics.

### Statistical Analysis

Statistical analyses were performed in collaboration with a statistician expert, using the Statistical Package for the Social Sciences (V.28, 2021, IBM Corporation, Armonk, New York, United States).

For each question of the ROPQ, the median and range were determined. The Kruskal–Wallis test was run to determine if there were differences in the performance scores between combat medics using an HMD and combat medics not using an HMD. Distributions of the performance scores for both groups were similar, as assessed by visual inspection. Therefore, differences between the Vuzix group, the RealWear group, and the control group were calculated using the median (range).

The one-way ANOVA test was used to interpret the total intervention time between the Vuzix group, RealWear group, and the control group. The Shapiro–Wilk test was run to see if it was possible to use the one-way ANOVA test. The Shapiro–Wilk test gave an insignificant result for the Vuzix group (*P* = .632) and an insignificant result for the control group (*P *= .507), which meant that these data were normally distributed. In the RealWear group, the Shapiro–Wilk test gave a significant result (*P *= .040). It was decided to still run the one-way ANOVA test as the sample sizes in the three groups were equal, and the one-way ANOVA test was considered a robust test.

For each question of the TUQ, the median and range were determined. The Kruskal–Wallis test was used to compare the median scores for each question between combat medics. Visual inspection showed that the distributions of the performance scores for both groups were similar. Therefore, differences between the Vuzix group, the RealWear group, and control group were calculated using the median (range).

## RESULTS

Nine combat medics were participated in this study. None of them had previously performed a fasciotomy or assisted a senior surgeon performing a fasciotomy.

**FIGURE 3.**
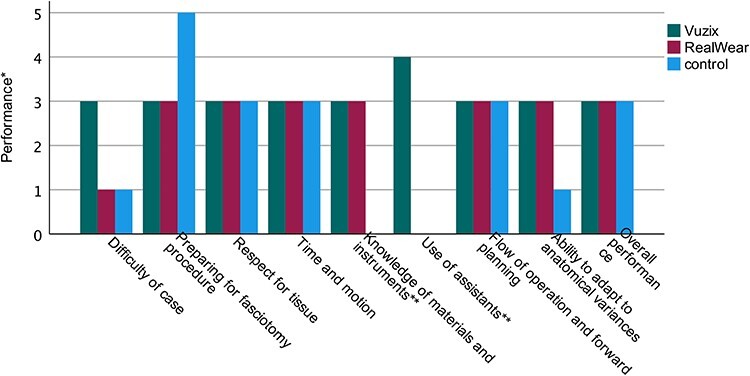
The median performance of combat medics.

The overall performance of combat medics in the Vuzix group, the RealWear group, and the control group scored 3 out of 5, which is considered competent (Fig. 3; Vuzix: median 3 [range 0], RealWear: median 3 [range 1], control: median 3 [range 1]). Combat medics in the Vuzix group and combat medics in the RealWear group scored 3 out of 5 in their ability to adapt to anatomical variances, which is a significantly better score compared to the control group (Fig. 3; Vuzix: median 3 [range 0], RealWear: median 3 [range 0], control: median 1 [range 0]; *P* = .018). All consulted senior surgeons noticed that combat medics using an HMD were more adequate in performing a fasciotomy than combat medics in the control group, who were less precise in execution and caused more tissue damage.

**FIGURE 4.**
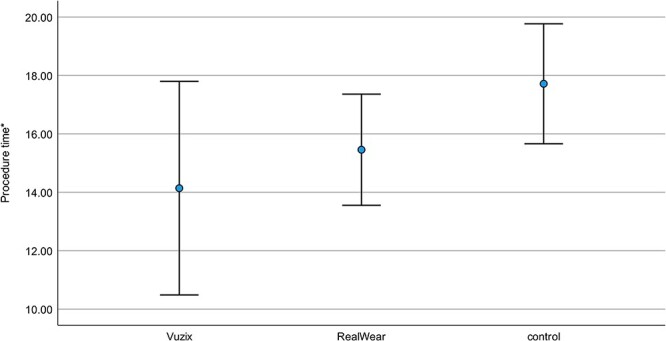
The mean procedure time (SD).

Both combat medics in the Vuzix group and the RealWear group finished the fasciotomy within acceptable intervention times and faster than the control group although not to a statistically significant degree (Fig. 4; Vuzix: mean 14:14 [SD 3:41], RealWear: mean 15:42 [SD 1:58], control: mean 17:45 [SD 2:02]; *P* = .340).

The overall satisfaction with both Vuzix M400 and RealWear HMT-1Z1 was 5 out of 7 (Supplementary 1; Vuzix: median 5 [range 0], RealWear: median 5 [range 1]; *P* = .317). Both HMDs would improve access to health care supervision, but combat medics favored the Vuzix M400 in this case (Supplementary 1; Vuzix: median 6 [range 1], RealWear: median 5 [range 0]; *P *= .034). The RealWear HMT-1Z1 scored significantly better on the ability to perform as expected by the combat medic using it (Supplementary 1; Vuzix: median 3 [range 1], RealWear: median 5 [range 0]; *P* = .034). Combat medics using the RealWear HMT-1Z1 had more optimal audio than combat medics using the Vuzix (Supplementary 1; Vuzix: median 2 [range 1], RealWear: median 6 [range 2]; *P* = .046). All combat medics using an HMD strongly agreed on using the HMD again (Supplementary 1; Vuzix: median 6 [range 2], RealWear: median 6 [range 1]; *P* = .637).

In both HMD groups, there were no episodes with poor quality of connection or connection failure. One combat medic closed off the HMD twice by accident, which was corrected quickly. This corresponds to the outcome of the TUQ, where combat medics agreed that they could easily and quickly recover a mistake they made using the HMD (Supplementary 1; Vuzix: median 4 [range 3], RealWear: median 5 [range 2]; *P* = .637). Four combat medics encountered volume and brightness setting issues that impaired the optimal functioning of the HMDs. This corresponds to the TUQ, in which combat medics disagreed on the ability of the HMD to give error messages that clearly told them how to fix problems (Supplementary 1; Vuzix: median 1 [range 3], RealWear: median 3 [range 2]; *P* = .369).

## DISCUSSION

In this feasibility study, we show that it is feasible to use an HMD to provide TM support from a consulted senior surgeon for combat medics when performing a fasciotomy. Combat medics using an HMD were considered competent in performing a fasciotomy with TM support after a standardized concise lecture. Their ability to adapt to anatomical variances was significantly better in comparison with the control group. Combat medics using an HMD were more adequate in performing a fasciotomy than combat medics in the control group, who were less precise in execution and caused more tissue damage. Fasciotomies by combat medics using an HMD were performed within acceptable intervention times, and combat medics using an HMD were faster than combat medics in the control group. Both the Vuzix M400 and the RealWear HMT-1Z1 devices were considered useful and had a good overall satisfaction score. Combat medics in both groups strongly agreed that they would use the HMD again in the future to receive TM support. The results of this study suggest that HMDs can be used to provide TM support for combat medics during tactical combat casualty care when performing life- and limb-saving procedures. Legislation was beyond the scope of this study; however, this is required before clinical implementation in austere environments.

For both groups, the standardized concise lecture was still fresh in memory. Anatomical variances of most anatomical structures at risk during a fasciotomy were taught in this lecture. The anatomical landmarks utilized to determine the correct size of the incision were explained using anatomical images and a video. This might explain why combat medics in the control group were also considered competent in performing a fasciotomy directly after the standardized concise lecture.

Further research should focus on the performance after at least 6 months to test if the HMD is applicable to provide TM support for low-experienced combat medics in saving training time and to improve the performance of a fasciotomy faster.

Our results correspond to the results reported by McPherson et al.,^14^ who showed that TM support enabled military physician assistants to perform a fasciotomy in a synthetic leg model. Other studies also show that it is feasible to use TM support to enable health care providers to safely perform a fasciotomy in remote environments.^15–17,23,24^ Unlike Park et al.,^15^ Rojas-Muñoz et al.,^17,23^ and Talbot et al.,^16^ our study population were combat medics. Including senior surgeons as participants could lead to a distorted view of the results as they might have previous experience with life- and limb-saving procedures. In this study, an AnubiFiX^®^-embalmed postmortem human leg was used, which might reflect the human anatomy better than a synthetic model, which was used by Liu et al.^24^ and McPherson et al.^14^ This study was set up to compare two devices between groups and a control group, and most previous studies^14,16,24^ neither test multiple devices nor had a control group.

No connection errors occurred in this study, as a stable Wi-Fi Protected Access 2 encrypted Wi-Fi connection was used. In future research, other connection options, such as 4G and satellite connection, should be tested as they are more likely to be used in austere environments. Cyber security is also a component that should be considered to protect the identity and geolocation of combat medics.

### Limitations

This feasibility study has several limitations. The study was conducted in a training OR, and austere environments can be different because of external factors. Future research should focus on the use of HMDs in (simulated) austere environments including the available surgical set as used by combat medics. Because of the small sample size, the generalizability of the results is limited. This is, however, not one of the main goals of a feasibility study. An international randomized controlled trial would be the study of choice, however very complex in setup because of the operational tasking of the combat medics. Another limitation of this study was the relatively low level of experience with the technical aspects of the HMD by both the consulted senior surgeon and the combat medics. As a result, the option for telestration, in which the consulted senior surgeon gives instructions using drawings on the screen of the HMD, was not sufficiently used. These technical issues can be optimized via both the HMD and the computer.

## CONCLUSION

This study shows that it is feasible to use an HMD to provide TM support from a consulted senior surgeon for combat medics when performing a fasciotomy. The results of this study suggest that TM support might be useful for combat medics during tactical combat casualty care when performing life- and limb-saving procedures.

## Supplementary Material

usad364_Supp

## Data Availability

The data that support the findings of this study are available on request from the corresponding author. All data are freely accessible.

## INSTITUTIONAL CLEARANCE

Institutional clearance was approved.

## References

[1] Strehle EM, Shabde N: One hundred years of telemedicine: does this new technology have a place in paediatrics? Arch Dis Child 2006; 91(12): 956–9.doi: 10.1136/adc.2006.099622. 17119071 PMC2082971

[2] Kim Y, Groombridge C, Romero L, Clare S, Fitzgerald MC: Decision support capabilities of telemedicine in emergency prehospital care: systematic review. J Med Internet Res 2020; 22(12): e18959.doi: 10.2196/18959. PMC7755537 33289672

[3] Mitrasinovic S, Camacho E, Trivedi N, et al: Clinical and surgical applications of smart glasses. Technol Health Care 2015; 23(4): 381–401.doi: 10.3233/THC-150910. 26409906

[4] Budrionis A, Augestad KM, Patel HR, Bellika JG: An evaluation framework for defining the contributions of telestration in surgical telementoring. Interact J Med Res 2013; 2(2): e14.doi: 10.2196/ijmr.2611. PMC3742399 23887078

[5] Yoon JW, Chen RE, Kim EJ, et al: Augmented reality for the surgeon: systematic review. Int J Med Robot 2018; 14(4): e1914.doi: 10.1002/rcs.1914. 29708640

[6] Butler FK Jr, Hagmann J, Butler EG: Tactical combat casualty care in special operations. Mil Med 1996; 161(Suppl 1): 3–16.doi: 10.1007/978-3-319-56780-8_1. 8772308

[7] Turner CA, Stockinger ZT, Gurney JM: Combat surgical workload in Operation Iraqi Freedom and Operation Enduring Freedom: the definitive analysis. J Trauma Acute Care Surg 2017; 83(1): 77–83.doi: 10.1097/TA.0000000000001496. 28426558

[8] Fisher AD, Naylor JF, April MD, Thompson D, Kotwal RS, Schauer SG: An analysis and comparison of prehospital trauma care provided by medical officers and medics on the battlefield. J Spec Oper Med 2020; 20(4): 53–9.doi: 10.55460/l8s6-cu4f. 33320313

[9] Mortensen SJ, Orman S, Serino J, Mohamadi A, Nazarian A, von Keudell A: Factors associated with development of traumatic acute compartment syndrome: a systematic review and meta-analysis. Arch Bone Jt Surg 2021; 9(3): 263–71.doi: 10.22038/abjs.2020.46684.2284. 34239953 PMC8221439

[10] Shadgan B, Menon M, O’Brien PJ, Reid WD: Diagnostic techniques in acute compartment syndrome of the leg. J Orthop Trauma 2008; 22(8): 581–7.doi: 10.1097/BOT.0b013e318183136d. 18758292

[11] Mortensen SJ, Vora MM, Mohamadi A, et al: Diagnostic modalities for acute compartment syndrome of the extremities: a systematic review. JAMA Surg 2019; 154(7): 655–65.doi: 10.1001/jamasurg.2019.1050. 31042278

[12] Ritenour AE, Dorlac WC, Fang R, et al: Complications after fasciotomy revision and delayed compartment release in combat patients. J Trauma 2008; 64(2 Suppl): S153–61; discussion S161–2.doi: 10.1097/TA.0b013e3181607750. 18376159

[13] Mackenzie CF, Bowyer MW, Henry S, et al: Cadaver-based trauma procedural skills training: skills retention 30 months after training among practicing surgeons in comparison to experts or more recently trained residents. J Am Coll Surg 2018; 227(2): 270–9.doi: 10.1016/j.jamcollsurg.2018.04.028. 29733906

[14] McPherson J, Kennedy C, Slobogean G, Hilsden R, Talbot M: Augmented-reality telementoring for leg fasciotomy: a proof-of-concept study. BMJ Mil Health 2023; 169(4): 355–8.doi: 10.1136/bmjmilitary-2021-001975. 35131888

[15] Park JP, Montreuil J, Nooh A, Martineau PA: Telemedicine-guided forearm emergency decompressive fasciotomy for compartment syndrome. J Telemed Telecare 2023; 29(1): 28–32.doi: 10.1177/1357633X20964359. 33070688

[16] Talbot M, Harvey EJ, Berry GK, et al: A pilot study of surgical telementoring for leg fasciotomy. J R Army Med Corps 2018; 164(2): 83–6.doi: 10.1136/jramc-2017-000817. 29018173

[17] Rojas-Munoz E, Cabrera ME, Lin C, et al: The system for telementoring with augmented reality (STAR): a head-mounted display to improve surgical coaching and confidence in remote areas. Surgery 2020; 167(4): 724–31.doi: 10.1016/j.surg.2019.11.008. 31916990

[18] Slieker JC, Theeuwes HP, van Rooijen GL, Lange JF, Kleinrensink GJ: Training in laparoscopic colorectal surgery: a new educational model using specially embalmed human anatomical specimen. Surg Endosc 2012; 26(8): 2189–94.doi: 10.1007/s00464-012-2158-y. 22286275 PMC3392504

[19] Theeuwes HP : A new model for training on human specimens in surgical-anatomical skills labs. Anat Physiol Biochem Int J 2017; 3(1): 0013–7.doi: 10.19080/APBIJ.2017.03.555604.

[20] Glarner CE, McDonald RJ, Smith AB, et al: Utilizing a novel tool for the comprehensive assessment of resident operative performance. J Surg Educ 2013; 70(6): 813–20.doi: 10.1016/j.jsurg.2013.07.009. 24209661

[21] Parmanto B, Lewis AN Jr, Graham KM, Bertolet MH: Development of the telehealth usability questionnaire (TUQ). Int J Telerehabil 2016; 8(1): 3–10.doi: 10.5195/ijt.2016.6196. PMC4985278 27563386

[22] Hajesmaeel-Gohari S, Bahaadinbeigy K: The most used questionnaires for evaluating telemedicine services. BMC Med Inform Decis Mak 2021; 21(1): 36.doi: 10.1186/s12911-021-01407-y. PMC7852181 33531013

[23] Rojas-Munoz E, Cabrera ME, Lin C, et al: Telementoring in leg fasciotomies via mixed-reality: clinical evaluation of the STAR platform. Mil Med 2020; 185(Suppl 1): 513–20.doi: 10.1093/milmed/usz234. 32074347

[24] Liu P, Li C, Xiao C, et al: A wearable augmented reality navigation system for surgical telementoring based on Microsoft HoloLens. Ann Biomed Eng 2021; 49(1): 287–98.doi: 10.1007/s10439-020-02538-5. 32504141

