# Supplementary material for: Telemedicine-Guided Two-Incision Lower Leg Fasciotomy Performed by Combat Medics During Tactical Combat Casualty Care: A Feasibility Study
Source: Mil Med. 2023 Sep 12;189(3-4):e645–51. doi: 10.1093/milmed/usad364 (PMC10898936; doi:10.1093/milmed/usad364)
Supplement: usad364_Supp [file usad364_supp.zip › Supplemental 1 version 1.2 20232508.pdf]

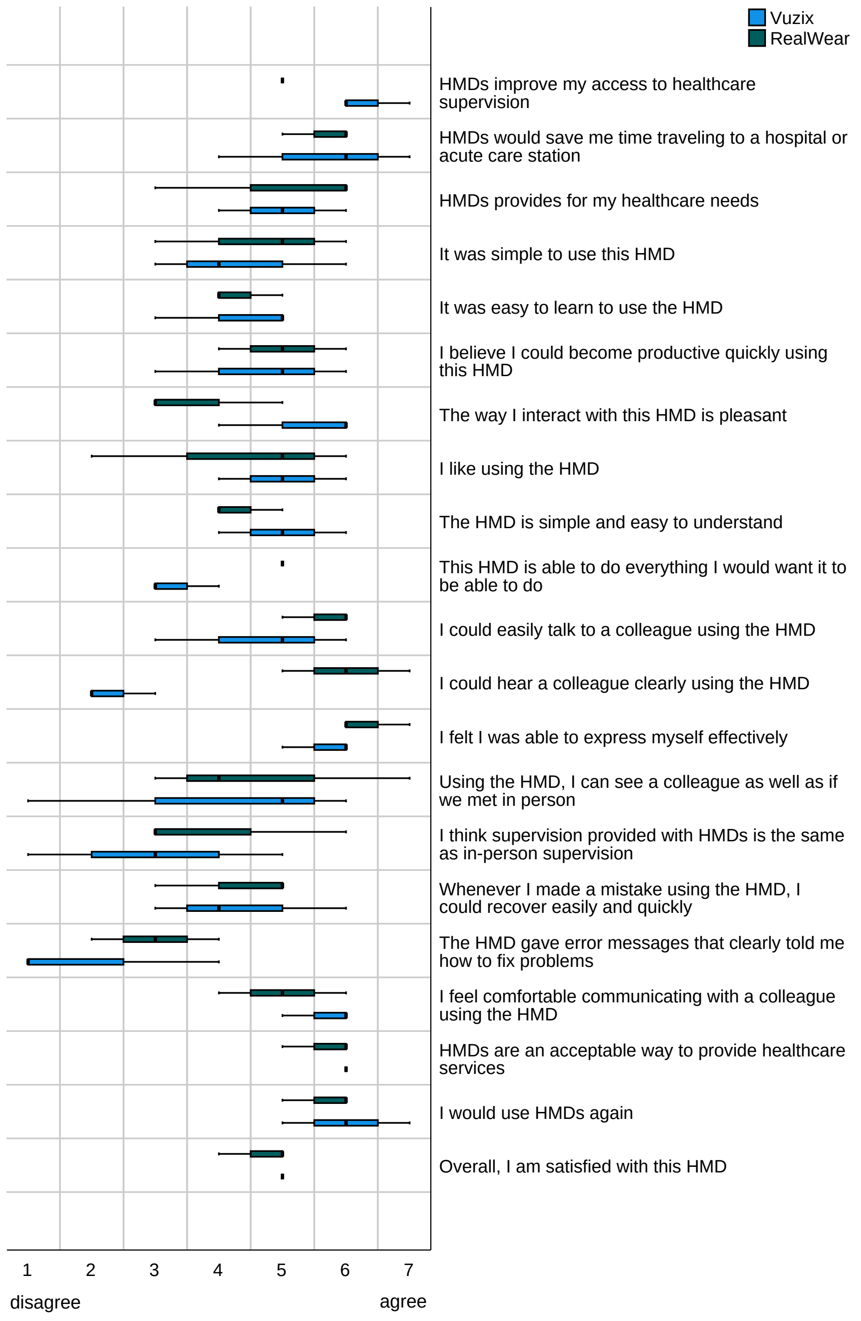


Supplemental 1: Telehealth Usability Questionnaire*
Scores are given in median (range)
* Kruskal Wallis test
